# Quantitative gait analysis under dual-task in older people with mild cognitive impairment: a reliability study

**DOI:** 10.1186/1743-0003-6-35

**Published:** 2009-09-21

**Authors:** Manuel Montero-Odasso, Alvaro Casas, Kevin T Hansen, Patricia Bilski, Iris Gutmanis, Jennie L Wells, Michael J Borrie

**Affiliations:** 1 Department of Medicine, Division of Geriatric Medicine, Parkwood Hospital, University of Western Ontario, London, ON, Canada; 2 Lawson Health Research Institute, London, ON, Canada; 3 Division of Geriatric Medicine, Hospital Universitario de Getafe, Madrid, Spain; 4 Specialized Geriatric Services, St Joseph's Health Care, London, ON, Canada

## Abstract

**Background:**

Reliability of quantitative gait assessment while dual-tasking (walking while doing a secondary task such as talking) in people with cognitive impairment is unknown. Dual-tasking gait assessment is becoming highly important for mobility research with older adults since better reflects their performance in the basic activities of daily living. Our purpose was to establish the test-retest reliability of assessing quantitative gait variables using an electronic walkway in older adults with mild cognitive impairment (MCI) under single and dual-task conditions.

**Methods:**

The gait performance of 11 elderly individuals with MCI was evaluated using an electronic walkway (GAITRite^® ^System) in two sessions, one week apart. Six gait parameters (gait velocity, step length, stride length, step time, stride time, and double support time) were assessed under two conditions: single-task (sG: usual walking) and dual-task (dG: counting backwards from 100 while walking). Test-retest reliability was determined using intra-class correlation coefficient (ICC). Gait variability was measured using coefficient of variation (CoV).

**Results:**

Eleven participants (average age = 76.6 years, SD = 7.3) were assessed. They were high functioning (Clinical Dementia Rating Score = 0.5) with a mean Mini-Mental Status Exam (MMSE) score of 28 (SD = 1.56), and a mean Montreal Cognitive Assessment (MoCA) score of 22.8 (SD = 1.23). Under dual-task conditions, mean gait velocity (GV) decreased significantly (sGV = 119.11 ± 20.20 cm/s; dGV = 110.88 ± 19.76 cm/s; p = 0.005). Additionally, under dual-task conditions, higher gait variability was found on stride time, step time, and double support time. Test-retest reliability was high (ICC>0.85) for the six parameters evaluated under both conditions.

**Conclusion:**

In older people with MCI, variability of time-related gait parameters increased with dual-tasking suggesting cognitive control of gait performance. Assessment of quantitative gait variables using an electronic walkway is highly reliable under single and dual-task conditions. The presence of cognitive impairment did not preclude performance of dual-tasking in our sample supporting that this methodology can be reliably used in cognitive impaired older individuals.

## Background

A large body of research has demonstrated an important interdependence between gait and cognition in elderly people noting that slow motor performance is associated with cognitive impairment and dementia [1-5]. Walking is a complex learned task that becomes automatic for most people from early childhood onwards. However, there is evidence that cognitive control of gait becomes increasingly important in older adults[6,7]. Since a seminal study demonstrated that the inability to maintain a conversation while walking is a marker for future falls in older adults[8], walking while performing a secondary task (dual-task paradigm) has become a classic way to assess the relationship between cognition and gait Because the dual-task paradigm is a realistic proxy for daily living activities that seniors may perform at home, it is growing in interest and application in clinical research settings.

Although previous studies have reported good test-retest reliability using electronic walkways while assessing gait in older people without performing a secondary task [9-11], the reliability of quantitative gait assessment under dual-task conditions among those with cognitive problems has not been established. One of the challenges faced when assessing cognitively impaired older adults is their potential inability to perform dual-tasking properly, thereby increasing measurement error. Therefore, our objective was to determine the reliability of quantitative gait assessment under both single and dual-task conditions in people with mild cognitive impairment (MCI) using a one-week space between assessments. This timeframe is appropriate for research purposes in this population, since gait variables are stable over short intervals. Establishing the reliability of quantitative gait assessment under dual-task conditions among those with cognitive problems is an important first step in validating this methodology for future longitudinal studies.

## Methods

### Subjects

Thirteen subjects were recruited from the Aging Brain and Memory Clinic (ABMC) at Parkwood Hospital, St. Joseph's Health Care, London, Ontario. Inclusion criteria were age 65 years and older, a diagnosis of MCI, and ability to communicate in English. Participants were diagnosed with MCI if they[12]: did not have dementia.[13], had objective memory impairment, experienced subjective memory symptoms corroborated by an informant, and had preserved activities of daily living (defined in our study as being able to perform basic and instrumental activities of daily living as evaluated by Lawton Brody Scale[14]). An additional MCI criterion was to have a Clinical Dementia Rating Scale (CDR) of 0.5[15].

Exclusion criteria included any objective gait disorder due to Parkinson's disease, previous stroke, clinical osteoarthritis in lower limbs joints, myopathy, or neuropathy as verified by a formal clinical examination. The presence of depressive symptoms, defined as a score ≥ 5/15 on the Geriatric Depression Scale[16], was also an exclusion criterion since depression may affect gait performance[16]. The Health Sciences Research Ethics Board at The University of Western Ontario approved the study. Subjects who consented to participate underwent a comprehensive medical examination by experienced geriatricians. Co morbidities, medications, falls in the previous 12 months, and fear of falling were recorded. Global cognitive status was assessed using the Mini Mental Status Exam (MMSE; scored 0-30)[17] and the Montreal Cognitive Assessment (MoCA; scored 0-30), a validated tool that was originally created to assist in the diagnosis of MCI[18]. A pattern of a low MoCA score (<26) with a normal MMSE score (>26) is associated with having MCI[18].

### Procedures

Each participant's gait performance was assessed using an electronic walkway system (GAITRite^®^) under a single (three trails) and a dual-task (three trails) condition per session over two sessions, spaced one week apart. Three trials per condition was found in the power analysis to be the optimum number of trials needed to obtain enough number of strides to be able to compute reliability assessment for the quantitative gait variables of interests[19]

The GAITRite^® ^system includes a portable electronic walkway mat (600 cm in length and 64 cm in width) for the automated measurement of spatiotemporal gait parameters. As participants walk along the mat, imbedded sensors are activated by the foot pressure and is deactivated when the pressure is released. A computer processed the footsteps, providing data for both spatial and temporal parameters. The following six gait variables were selected based on their clinical relevance and their reported association with cognitive function in previous aging studies[5,16,20,21]: gait velocity (cm/s), step length (cm), stride length (cm), step time (sec), stride time (sec), and double support time (sec). Gait parameters were recorded using only the footprint of the participants, thereby eliminating the need for external sensors attached to the body or lower limbs that may interfere with the gait performance.

GAITRite^® ^system resolution is in milliseconds for time parameters and in millimeters for distances and lengths parameters. The mat was located in a well-lit, 10-meter long hallway with starting and ending limits marked one meter from the mat to avoid recording acceleration and deceleration phases.

### Gait assessments

Prior to the trials, participants were giving standardized instructions and a visual demonstration. Then, participants were asked to perform three single-task trials and three dual-task trials. The single task trials consisted of walking the length of the mat at self-selected pace (sG). For the dual-task trials, participants walked the length of the mat while counting backward from one hundred by one aloud (dG). This dual-task condition was selected based on previous research which demonstrated that counting backwards requires both working memory and attention[22]. There was no instruction to prioritize either gait or cognitive task; however, if a participant stopped either task during the trial they were prompted to continue. Allowing both aspects to vary, gait and cognitive task, has previously been shown to better represent the dynamics of daily living tasks of older adults[23,24].

### Data acquisition of the quantitative gait variables

GAITRite software Version 3.8 was used to process the footstep data using the settings for light and short footsteps as individuals with MCI may be more likely to slow down or hesitate while dual-tasking. If a participant's first or last footstep did not fall completely within the active area of the walkway these footstep were manually removed from the recorded walk. Further, to minimize environmental variability, evaluations were conducted on the same weekday (± 1 day) and at the same time of day, with participants instructed to wear the same pair of shoes for both sessions.

### Statistical analysis

Baseline characteristics and gait parameters were summarized using either means and standard deviations, or frequencies and percentages, as appropriate. For each gait parameter and for both conditions, the mean of the three trials was used in the analysis. Three trials was found to be the optimum number of trials needed to obtain enough number of strides to be able to compute reliability assessment for the quantitative gait variables of interests[19]. Comparisons between means obtained during sG and dG conditions were performed using a paired t-test. To quantify gait variability under both single and dual-task conditions, the coefficient of variation[25] (CoV = SD/mean*100) of each gait variable was calculated at each time point.

The Intraclass Correlation Coefficient (ICC), based on a two-way random effects analysis of variance, was used to quantify test-retest reliability. To interpret ICC values we used bench marks suggested by Cicchetti (if ICC<0.40, the level of clinical significance is "poor;" between 0.40 and 0.59 is "fair;" between 0.60 and 0.74 is "good;" and between 0.75 and 1.00 the level of clinical significance is "excellent."[26]). We preferred ICC to evaluate test-retest reliability over a standard correlation analysis because ICC accounts for differences between data sets by using analysis of variance. The level of statistical significance was set at 0.05 and analyses were conducted using SPSS version 15.0 (SPSS Inc., Chicago IL).

## Results

Of the 13 individuals recruited from the ABMC, 2 were excluded due to gait-affecting comorbidities yielding a final study group of 11 participants. Demographic and medical characteristics are summarized in Table 1. In brief, they were five males and six females, with a mean age of 76.6 years (SD = 7.3). They were high functioning in terms of instrumental activities of daily living with a mean Lawton-Brody = 7.18 out of 8 (SD = 1.06) with higher scores indicating better functionality Four participants had experienced a fall in the preceding 12 months. Global cognitive functioning pattern was consistent with the diagnosis of MCI[18].

**Table 1:** Characteristics of the participants (n = 11).

**Characteristics**	**Mean (SD)**
Age	76.6 (7.3)
Gender - Female: n (%)	6 (54%)
History of at least 1 fall in last 12 months: n (%)	4 (36%)
Body Mass Index (BMI)	25.8 (4.4)
Years of education	14.1 (3.4)
Functional capacity	
Lawton-Brody Score IADLs (max. score = 8)	7.18 (1.1)
Global cognition	
MMSE Score (0-30)	28 (1.6)
MoCA score (0-30)	22.8 (1.2)
Gait Velocity (cm/s)	
Single Gait Velocity (sGV)	119.1 (20.2)
Counting Gait Velocity (cGV)	110.8 (19.8)

***Note: ***SD = Standard deviation; IADL = Instrumental Activities of Daily Living; MMSE = Mini-Mental State Examination; MoCA = Montreal Cognitive Assessment.

At baseline assessment (week 1), mean gait velocity under single-task conditions (sG) was significantly faster than the gait velocity under dual-task conditions (dG) (sG = 119.11 vs. dG = 110.88 cm/s, p = 0.005, Table 2). This mean decrement of 8.23 cm/s, a seven-percent change, is considered a clinically significant change in gait velocity[6,27]. At Week 2, although gait velocity decreased under dG conditions, the difference in velocity was not statistically significant (sG: 113.18 vs. dG: 111.84 cm/s, p = 0.579).

**Table 2:** Overview of the Gait Parameters for Single (sG) and Dual-Task (dG) Conditions.

	**Single Task Gait (sG)**				
	**Week 1**		**Week 2**		

**Gait Variable**	**Mean (SD)**	**CoV**	**Mean (SD)**	**CoV**	**ICC (95% CI)**

Gait velocity (cm/s)	119.11 (20.2)	16.96	113.18 (15.27)	13.49	0.87 (0.52 - 0.97)
Step length (cm)	65.88 (12.03)	18.26	64.04 (10.66)	16.65	0.97 (0.88 - 0.99)
Stride length (cm)	132.07 (24.14)	18.28	128.52 (21.22)	16.51	0.97 (0.89 - 0.99)
Step time (s)	0.55 (0.04)	7.27	0.57 (0.04)	7.02	0.87 (0.51 - 0.96)
Stride time (s)	1.10 (0.07)	6.36	1.13 (0.08)	7.08	0.86 (0.49 - 0.96)
Double support time (s)	0.31 (0.04)	12.90	0.32 (0.04)	12.50	0.80 (0.25 - 0.95)

	**Dual Task Gait (dG)**				

	**Week 1**		**Week 2**		

**Gait Variable**	**Mean (SD)**	**CoV**	**Mean (SD)**	**CoV**	**ICC (95% CI)**

Gait velocity (cm/s)	110.88 (19.76)	17.82	111.84 (17.48)	15.63	0.93 (0.75 - 0.98)
Step length (cm)	65.48 (12.62)	19.27	64.70 (10.49)	16.21	0.97 (0.88 - 0.99)
Stride length (cm)	131.49 (25.25)	19.20	126.86 (20.95)	16.51	0.97 (0.88 - 0.99)
Step time (s)	0.59 (0.07)	11.86	0.58 (0.07)	12.07	0.96 (0.86 - 0.99)
Stride time (s)	1.18 (0.13)	11.02	1.16 (0.13)	11.21	0.96 (0.85 - 0.99)
Double support time (s)	0.34 (0.06)	17.65	0.34 (0.05)	14.71	0.95 (0.82 - 0.99)

**Figure 1:**
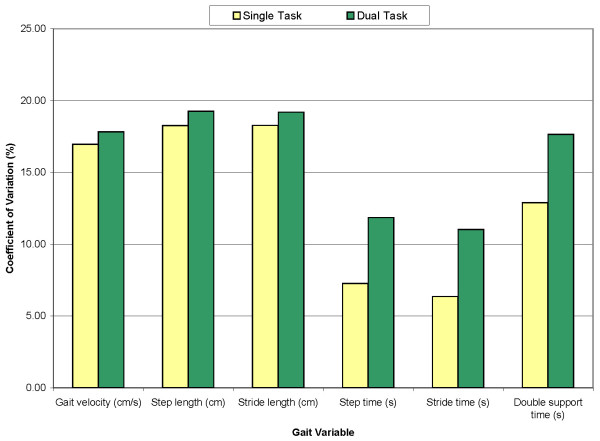
**Gait variability under single and dual tasks for the six gait variables assessed at baseline**.

Gait variability results are expressed as CoV in Table 2. Of the six parameters analyzed, only step and stride time have significantly increased during dual-task condition and with considerable stride-to-stride variability. In fact, the CoV for stride time increased from 6.36 on sG to 11.02 on dG in week-one assessment. As shown in Figure 1, under dual task conditions, there was an increase in the CoV for the variables related to time: step, stride, and double support time. For these time-related variables, the CoV increased by 63.13%, 73.27%, and 36.82% respectively from sG to dG conditions, while the CoV for the remaining gait variables increased by less than 6%.

Reliability results are shown in Table 2. Under single task conditions, re-test reliability was excellent with the ICCs for six gait variables being 0.83 or higher with the exception of double support time, which was 0.80. Reliability results under dual-task conditions were also excellent with ICCs for the six gait measures were 0.93 or higher.

## Discussion

This study established the test-retest reliability of gait assessment under single and dual-task conditions in older adults with MCI. There is excellent reliability in both conditions over a one-week time span. Reliability of gait assessment in older adults has been reported previously[11,20,28]; however, to our knowledge, this is the first report which determined the reliability under dual-task conditions in mildly cognitively impaired older people. Due to the growing importance of dual-task paradigm in current research of cognitive decline, falls, and dementia [29-31], our results are of particular relevance since the presence of cognitive impairment in our population did not preclude performance of dual-tasking while gait assessment.

The ICCs for the six variables analyzed under single and dual-task were higher than 0.75, showing excellent clinical reliability for the assessment of these spatial and temporal gait parameters.

One interesting finding of our study is that the time required to perform steps and strides significantly increased under the dual-task condition. This is consistent with previous studies, which have demonstrated that these parameters have a greater dependence on brain cortical control than other gait parameters[25,31]. In addition, step and stride time showed a greater variability under dual-task conditions when compared with the other parameters analyzed (Table 2, Figure 1). This finding is particularly relevant given that gait variability under dual-tasking has been demonstrated to be an early predictor of future falls[8] In our sample of people with Mild Cognitive Impairment, gait variability significantly increased under dual-task conditions. While gait variability is minimal under dual-task conditions for the general population, high gait variability is associated with Parkinson disease, Alzheimer's disease and other dementias, a variety of types of dementias and is a predictor of future falls[25,30,31]. Since our results confirm the reliability of this assessment in persons with MCI, this type of assessment may be an effective early measure of detecting individuals with MCI who are at higher risk of future falls. Increased gait variability represents a more unstable walking pattern with less rhythmicity. We postulate that this altered gait pattern is a type of "arrhythmia" of the gait and may correlate to other health outcomes such as future dementias, falls or other comorbidities.

Particularly, our results provide support to apply this methodology in people with cognitive problems. Although gait has long been considered as primarily an automatic motor task, emerging evidence suggests that this view may be overly simplistic[32]. Cortical brain control may play a key role in the regulation of even routine walking, specifically through attention. Attention is a necessary cognitive resource for maintaining normal walking and attentional deficits are independently associated with postural instability, impairment in performing daily living activities, and future falls[24]. Specifically, dual-tasking cost has been traditionally related to the prefrontal cortical regions[33]. These brain regions are crucially involved in the mediation of the division of attention and executive function. Functional neuroimaging studies showed correlations between dual-task performance with increase activity in prefrontal areas, cingulate, parietal and premotor areas[34,35]. Therefore, we postulate that those regions may have a control on gait in older individuals. In line with previous studies, our results support the hypothesis that occupying these areas with concurrent cognitive processing (dual-tasking) may result in a brain resource limitation that affects gait in people with MCI.

Strengths of our study include the use of a well-defined population that met strict criteria for MCI, and the use of established and validated measures of cognition using a sophisticated quantitative gait assessment. The small sample size of our study, however, is a potential limitation, as it may not represent the full spectrum of MCI population. Despite these limitations, this study provides evidence that quantitative gait analysis while dual-tasking among older adults with MCI is a reliable methodology.

## Conclusion

In older adults with MCI, assessment of quantitative gait variables using an electronic walkway was highly reliable under single and dual-task conditions. In line with previous studies conducted in elderly with normal cognition, variability of time-related gait parameters increased while dual-tasking in our sample. The presence of cognitive impairment did not preclude performance of dual-tasking gait assessment in our sample; therefore, this methodology can be reliably used in older people with MCI.

## Competing interests

The authors declare that they have no competing interests.

## Authors' contributions

Study concept and design (MMO and MB), acquisition of subjects and data (MMO, AC, MB, JW, PB), data analysis (MMO, KTH, IG), preparation of the manuscript (MMO, KTH), and critical review of the manuscript (MMO, KTH, AC, MB, JW, IG). All authors read and approved the final manuscript.
